# Corrosion and Mechanical Properties of Q500 qENH Steel in Simulated Plateau Environment

**DOI:** 10.3390/ma18163923

**Published:** 2025-08-21

**Authors:** Yanchen Liu, Xin Liu, Tao Lan, Zexu Li, Guangjie Xing, Shuailong Song

**Affiliations:** 1 School of Civil Engineering, Qingdao University of Technology, Qingdao 266520, China; yanzhao3255@163.com (Y.L.);; 2 CSSC International Engineering Co., Ltd., Beijing 100121, China; 3 School of Civil Engineering, Xian University of Architecture & Technology, Xi’an 710055, China

**Keywords:** weathering steel, periodic immersion corrosion, tensile mechanical properties, high-altitude corrosive

## Abstract

In high-altitude corrosive environments, weathering steel is widely applied due to its excellent corrosion resistance. However, the welded joint regions, where the chemical composition and microstructure undergo changes, are susceptible to the corrosion-induced degradation of mechanical properties. This study investigates the corrosion–mechanical synergistic degradation behavior of a 16 mm thick Q500 qENH base metal and its V-type and Y-type welded joint specimens. Periodic immersion corrosion tests were conducted to simulate plateau atmospheric conditions, followed by mechanical performance evaluations. Corrosion metrics—including corrosion rate, cross-sectional loss, penetration depth, and corrosion progression speed—were analyzed in relation to mechanical indicators such as the fracture location, yield load, ultimate load, yield strength, and tensile strength at varying exposure durations. The results indicate that the corrosion process exhibits distinct layering, with a two-stage characteristic of rapid initial corrosion followed by slower progression. Welded joints consistently exhibit higher corrosion rates than the base metal, with the rate difference evolving nonlinearly in an “increase–decrease–stabilization” trend. After corrosion, the mechanical performance degradation of welded joint specimens is more severe than that of base metal specimens.

## 1. Introduction

Under the national strategic of promoting infrastructure with “long service life and low maintenance” objectives, the corrosion resistance of steel structural materials has become a critical bottleneck limiting the sustainable development of civil engineering. Q500 qENH weathering steel achieves both corrosion resistance and high-strength performance through alloying element addition strategies, providing a revolutionary solution for modern steel structural engineering. Compared with conventional steels, Q500 qENH weathering steel significantly enhances the weathering index by precisely controlling the carbon content below the critical threshold and incorporating trace elements such as Cu, Cr, and Ni [1,2]. This results in a service life extension of more than three times under typical industrial atmospheric conditions.

Microstructural characterization reveals that the inner surface of Q500 qENH weathering steel forms a dense rust layer containing over 75% α-FeOOH, which chemically bonds with the substrate matrix to establish an efficient corrosion barrier. This mechanism fundamentally transforms the conventional passive anti-corrosion paradigm for structural steels. Asami and Kikuchi [3] reported that the combined effects of Cu redox cycling, Cr-rich passive film self-healing, and Si-based gel filling significantly enhance corrosion resistance under complex atmospheric conditions.

With advances in materials science and engineering disciplines, the content and composition of alloying elements in weathering steels have been optimized, significantly enhancing their corrosion resistance in bridges exposed to harsh environments such as high-altitude and marine conditions [4,5,6,7,8,9,10]. Furthermore, extensive research on weathering steel has enabled the optimization of the content and composition of alloying elements, significantly enhancing its corrosion resistance, applicability, and cost-effectiveness [11,12,13,14,15,16].

Current research on the corrosion mechanisms of weathering steel primarily focuses on conventional atmospheric conditions [17,18]. However, the distinctive environmental parameters of China’s plateau regions—including low atmospheric pressure, significant diurnal temperature variations, intense ultraviolet radiation, and drastic wet–dry cycles [19,20]—may induce corrosion behaviors markedly different from those in traditional environments. For instance, large diurnal temperature fluctuations can lead to the formation of temporary liquid films on exposed steel surfaces, resulting in a liquid-film-like effect that accelerates corrosion. Weathering steels are frequently employed in bridge construction under harsh atmospheric environments, such as high-altitude cold regions. Researchers have simulated the corrosion behavior of Q345 qDNH bridge weathering steel in industrial atmospheric corrosion environments [21,22]. With a prolonged exposure time, the inner corrosion product layer thickness on Q345 qDNH steel surfaces significantly increases. The enrichment of Ni, Cr, and Cu oxidation products within the inner layer enhances the compactness and stability of the oxide film, thereby improving the material’s corrosion resistance.

To investigate the mechanical properties and performance degradation of weathered steel after corrosion, some scholars have conducted relevant research. Li Yongliang et al. [23] analyzed the effect of post-rolling cooling modes on the mechanical property variations of weathering steel for container applications. The results indicate that the transition of strengthening mechanism from pearlite strengthening to ferrite fine-grain strengthening is the key to the improvement of mechanical performance. Luo et al. [24] conducted a comparative study on the mechanical properties of Q345B and 09CuPCrNi-A weathering steels under both ambient and post-corrosion conditions. The experimental results demonstrated that 09CuPCrNi-A weathering steel exhibits superior corrosion resistance and more balanced comprehensive mechanical properties. Wang et al. [25] conducted atmospheric exposure tests to analyze the mechanical properties of weathered steel after corrosion. The results demonstrate that Cl plays a dominant role in the initial corrosion stage, leading to significant degradation in the mechanical performance of the corroded weathering steel.

Existing research has demonstrated that environmental factors significantly influence the mechanical properties of weathering steel. Although progress has been made in corrosion behavior studies of weathering steel, notable gaps remain in complex plateau environments: systematic research on corrosion kinetics and the service life evolution of Q500 qENH weathering steel is lacking. The fracture morphology and failure characteristics of the specimens have not been clearly elucidated, and the fracture morphology and failure characteristics of corroded joints have not been clearly elucidated. To address these gaps, this study employs cyclic immersion corrosion testing combined with mechanical characterization to evaluate the corrosion behavior, kinetics, and tensile performance of both Q500 qENH base metal and welded joint specimens. By integrating corrosion and mechanical data, this work aims to elucidate regional degradation patterns and establish a theoretical foundation for the weld design and parameter optimization of Q500 qENH weathering steel in complex, pollution-intensive plateau environments.

## 2. Experimental Study on Accelerated Corrosion Testing of Weathering Steel Specimens in Laboratory

### 2.1. Design and Parameters of Corrosion Specimens

This study presents an experimental investigation into the corrosion behavior and post-corrosion tensile properties of Q500 qENH weathering steel under complex atmospheric pollution conditions representative of plateau regions.

**Figure 1:**
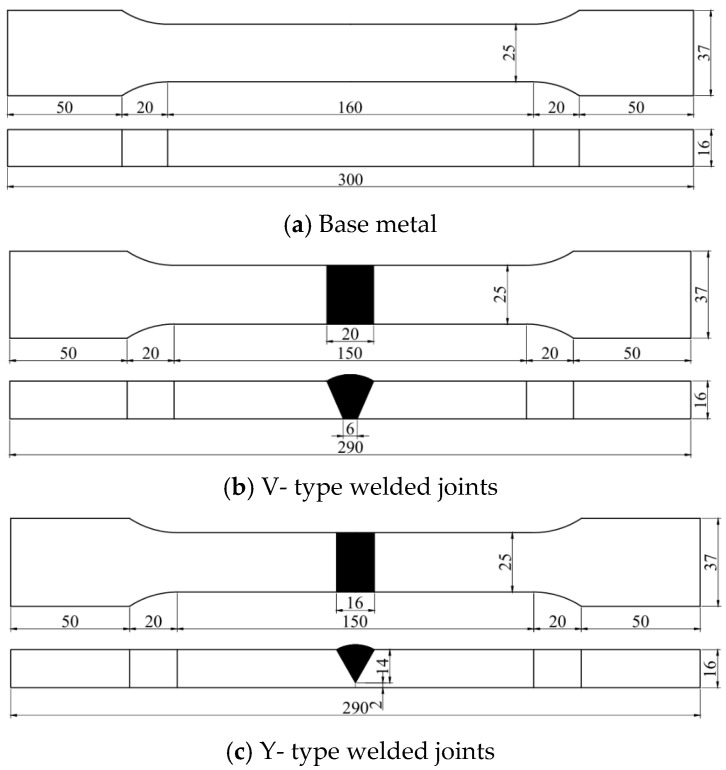
The shape and geometric dimensions of the specimen.

**Figure 2:**
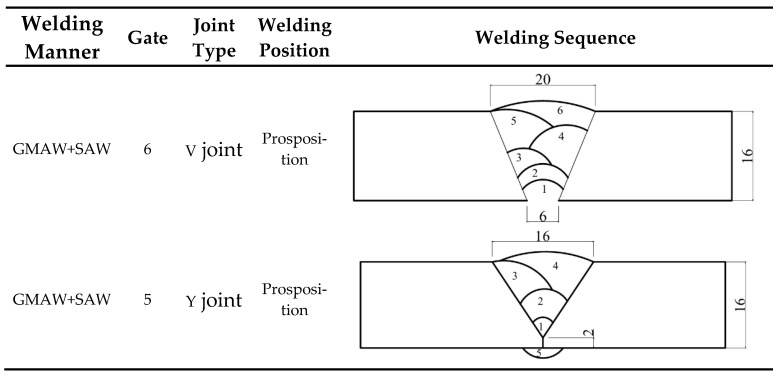
Welding joint size and welding sequence of the specimen.

All specimens were precisely machined in the factory using wire cutting technology. After the cutting process, the entire surface area of each specimen was thoroughly inspected to eliminate those with defects such as pores or cracks. The base material used in the tests of this paper was 16 mm thick Q500 qENH weather-resistant steel, newly developed by Nanjing Iron and Steel Co., Ltd., Nanjing, China. The welding joints employed were 1.2 mm diameter JWER60NHQ solid wire and 4 mm diameter JWS60NHQ submerged arc welding wire, was performed by China Railway Baoji Bridge Group Co., Ltd., Baoji, China. The test adopted a combined welding approach using two methods: Gas Metal Arc Welding (GMAW) and Submerged Arc Welding (SAW). Among these, GMAW (Gas Metal Arc Welding) was performed manually and SAW (Submerged Arc Welding) was completed using an MZ-1000 automatic submerged arc welding machine by Shanghai Huashen Electric Welding Machine Factory, Shanghai, China. The morphology and geometric dimensions of the corrosion specimens are illustrated in Figure 1, with their chemical composition detailed in Table 1. The chemical composition of the welding wire used for the welded joints is presented in Table 2. The welding joint size and welding sequence of the specimen are shown in Figure 2.

### 2.2. Grouping of Corrosion Specimens

A comparative analysis was conducted on the corrosion behavior between uncorroded specimens and three types of specimens (base metal, V-type welded joints, and Y-type welded joints) of Q500 qENH weathering steel under three corrosion cycles (9 d, 18 d, 27 d). Three parallel specimens were prepared for each type to ensure data reliability, with detailed grouping information presented in Table 3.

### 2.3. Test Methods

#### 2.3.1. Corrosion Testing Equipment and Simulation

**Figure 3:**
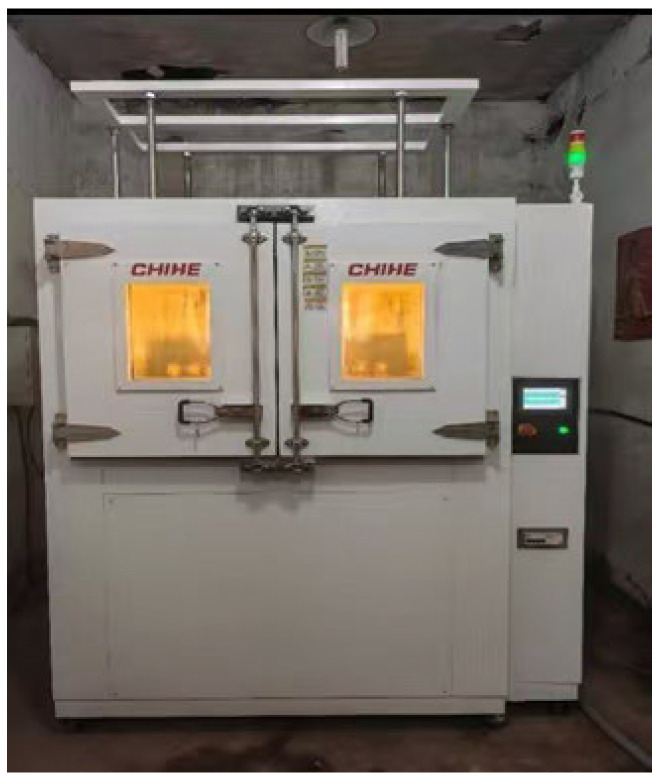
Periodic wetting corrosion test chamber.

To investigate the corrosion resistance of Q500 qENH weathering steel in the complex atmospheric pollution environment of plateau regions, cyclic immersion corrosion tests with accelerated corrosion effects were employed to simulate the plateau conditions for Q500 qENH weathering steel. In recent years, with the increasing emission of industrial pollutants, SO_2_ concentrations have shown a gradual upward trend in the Sichuan and Qinghai regions [26]. As SO_2_ is the corrosion yield of steel, this study employed a CHFL-360 cyclic immersion corrosion testing apparatus by Wuxi China Testing Instrument Co., Ltd., Wuxi, China for relevant experiments, as illustrated in Figure 3. The test medium employed was a sodium bisulfite (NaHSO_3_) solution with a controlled concentration of (2.0 ± 0.05) × 10^−2^ mol/L. The solution pH was monitored using a pH meter and maintained within the range of 4.4–4.8. To ensure the stability of the medium concentration during testing, an equivalent concentration compensation solution was prepared for dynamic replenishment. The NaHSO_3_ solution, in combination with the weathering steel, facilitated electrochemical corrosion to accelerate the corrosion process, thereby simulating the corrosive effect of HSO_3_^−^ (an oxidation product of SO_2_ in actual highland atmospheres) on the weathering steel specimens. The test chamber was set to a temperature of 45 °C and a relative humidity of 70% RH. Each wet–dry cyclic corrosion test (CCT) consisted of a 12 min immersion phase followed by a 48 min drying phase [27].

After cyclic immersion, the specimens were subjected to a hybrid derusting method. First, a nylon brush was used to remove loose outer-layer corrosion products. Subsequently, the specimens were immersed in an acidic derusting agent to chemically eliminate the dense inner-layer corrosion products. Finally, the derusted specimens were further cleaned via sequential immersion in distilled water and acetone solution.

#### 2.3.2. Tensile Test

**Figure 4:**
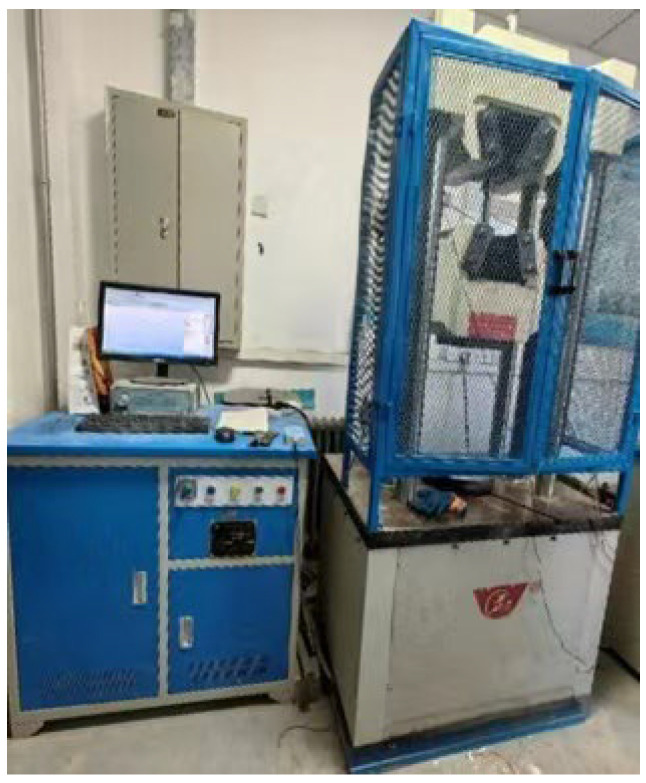
Universal testing machine.

All tensile specimens in this experiment were tested using the EHC-3100 microcomputer-controlled electro-hydraulic servo universal testing machine by Wuxi Jianyi Instrument & Machinery Co., Ltd., Wuxi, China (Figure 4), with a loading rate of 2.40 mm/min. To mitigate the effects of clamping slippage and non-uniform stress distribution caused by corrosion layers, all specimens underwent combined mechanical–chemical rust removal prior to tensile testing, in accordance with the standard [28] “Corrosion of metals and alloys-Removal of corrosion products from corrosion test specimens.”

## 3. Parameter Analysis of Weathering Steel After Corrosion

### 3.1. Analysis and Comparison of Specimen State After Corrosion

#### 3.1.1. Macroscopic Corrosion Morphology

**Figure 5:**
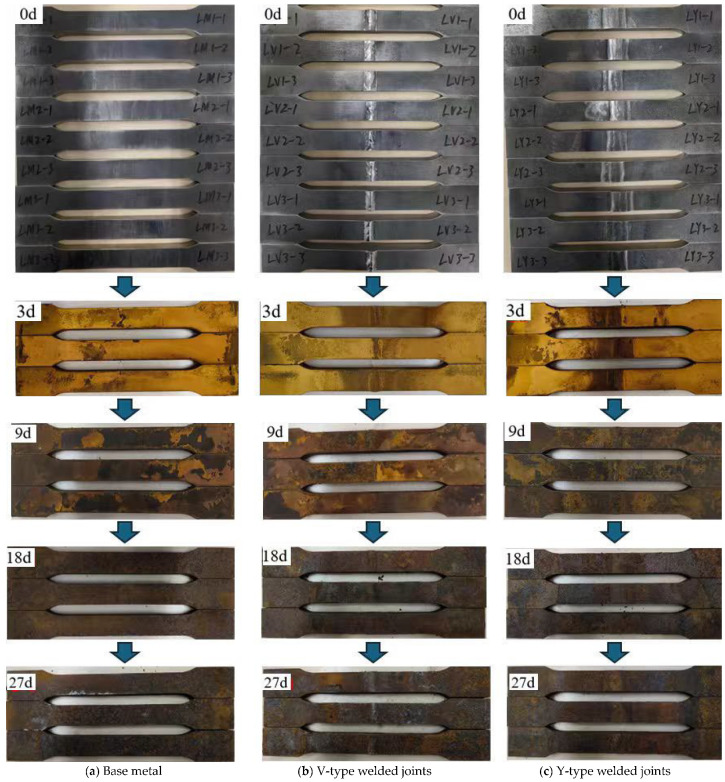
Macroscopic corrosion morphology of corrosion specimen surface.

A staged sampling method was used to observe the macroscopic corrosion morphology of the base metal, V-type, and Y-type welded joint specimens at different corrosion stages: day 0 (0 d), day 9 (9 d), day 18 (18 d), and day 27 (27 d). With prolonged corrosion exposure, a stratified oxide product gradually formed on the specimen surfaces, exhibiting a composite rust layer structure consisting of an outer rust layer and an inner rust layer. The outer rust layer typically displayed five to six distinct sublayers. Under the influence of thermal air circulation, the outer rust layer underwent continuous exfoliation while the inner rust layer progressively densified. Figure 5 presents the macroscopic corrosion morphologies of specimens at various stages.

#### 3.1.2. Stage Corrosion Characteristics

Visual inspection revealed that as corrosion progressed, black oily corrosion products initially formed on the surface, followed by the gradual development of yellowish-brown rust layers accompanied by granular iron oxide precipitation. Compared to the base metal, the weld zone of the welded joint specimens exhibited darker-colored corrosion products with more pronounced corrosion features. After 9 days of corrosion, the yellowish-brown rust layers on both the base metal and welded joint specimens began to degrade, with the appearance of light-brown clustered deposits arranged in stratified patterns. These deposits exhibited dense intergranular stacking, and dark-brown spots appeared locally. A notable characteristic was the significant increase in overall rust layer thickness, accompanied by distinct stratification between the inner and outer rust layers. By 18 days, the rust layers had completely transitioned to dark-brown coloration. The outer rust layers on both the base metal and welded joint specimens started to spall, revealing the inner rust layers in some localized areas. At 27 days, the macroscopic corrosion morphology of both specimen types remained largely consistent with the 18-day observations. However, the distinguishing feature was the intensified spalling of rust layers, with inner rust layers becoming exposed in most areas. The compactness of the inner rust layers had significantly improved, and the corrosion rate appeared to approach stabilization.

#### 3.1.3. Microstructural Corrosion Morphology

**Figure 6:**
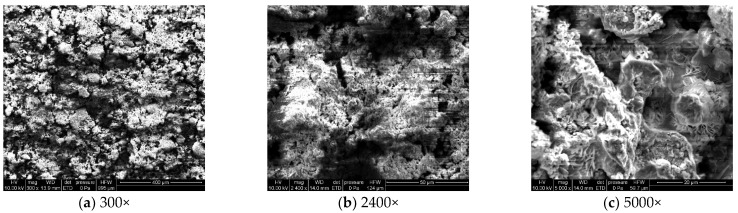
Microscopic morphology of the uncorroded specimen.

**Figure 7:**
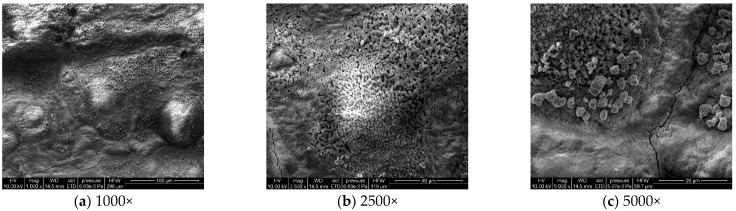
Microscopic morphology of specimens after 9.

**Figure 8:**
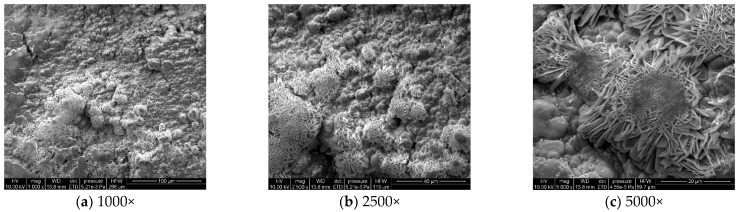
Microscopic morphology of specimens after 18.

**Figure 9:**
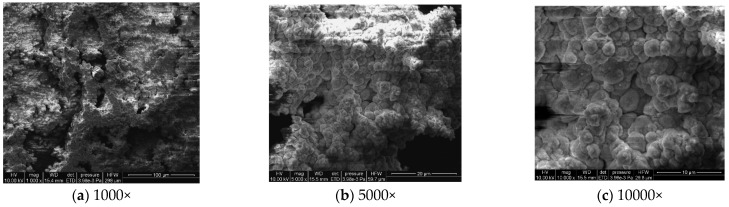
Microscopic morphology of specimens after 27.

To analyze the microscopic morphology of the specimens at different corrosion stages, a scanning electron microscope (SEM) was used to observe the surface of the specimens. The magnification was progressively increased. Figure 6 shows the microscopic morphology of the uncorroded specimen, while Figure 7, Figure 8 and Figure 9 show the microscopic morphology of specimens after 9, 18, and 27 days of corrosion, respectively.

During the corrosion process, the specific phases within the rust layer can be preliminarily identified by their microscopic morphology. Research has shown that needle-like γ-FeOOH, rose petal-like β-FeOOH, and cotton ball-like α-FeOOH are common phases in rust layers [29]. In the initial stages of corrosion, needle-like γ-FeOOH, an early corrosion product, forms on the surface of the uncorroded specimen. As corrosion progresses, rose petal-like β-FeOOH appears in the mid-stage. In the late stage of corrosion, the rust layer is predominantly composed of cotton ball-like α-FeOOH. These morphological transitions indicate that the composition of the corrosion products evolves over time, with the early formed γ-FeOOH gradually transforming into the more stable α-FeOOH.

### 3.2. Comparison of Parameters After Corrosion

**Figure 10:**
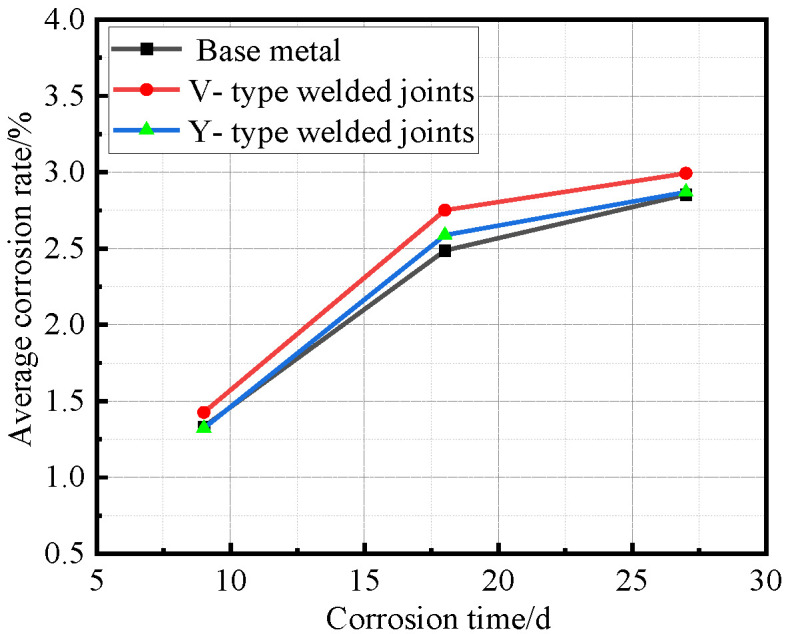
Relationship between average corrosion rate and corrosion time.

**Figure 11:**
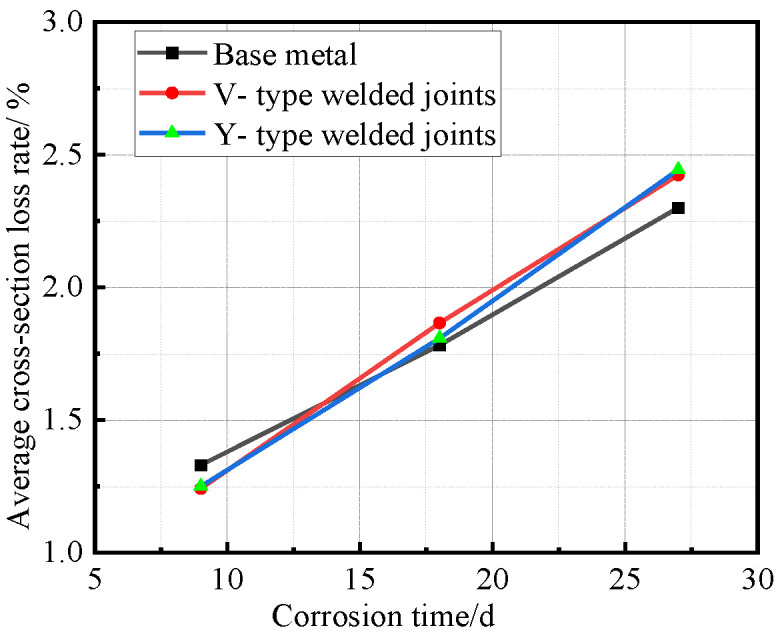
Relationship between average cross-section loss rate and corrosion time.

**Figure 12:**
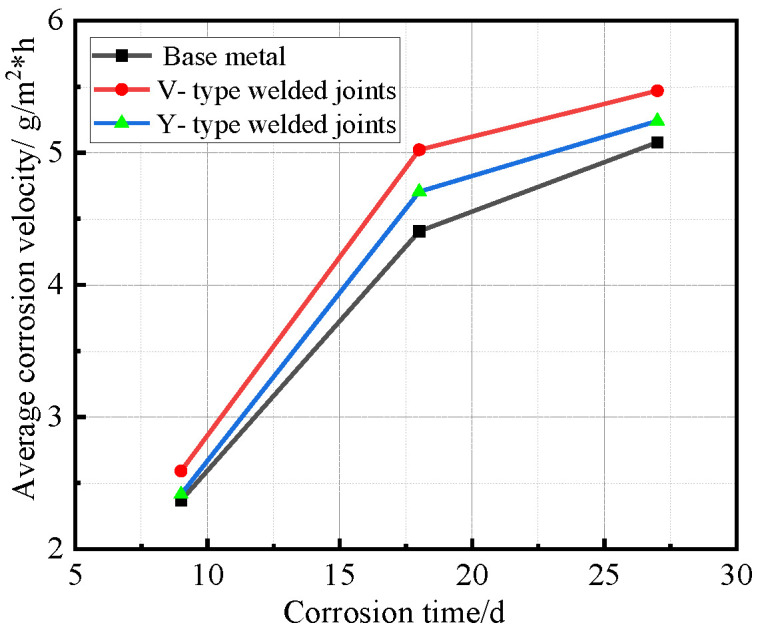
Relationship between average corrosion velocity and corrosion time.

After all Q500 qENH weathering steel specimens completed four corrosion cycles and reached the predetermined corrosion duration, they were extracted, cleaned, and weighed. Based on the recorded mass loss and dimensional data, the corrosion rate, corrosion velocity, and corrosion depth were quantitatively calculated. The corrosion rate data versus time for the base metal specimens, V-type welded joint specimens, and Y-type welded joint specimens of Q500 qENH weathering steel were fitted to establish their respective average corrosion rate curves, average section loss rate curves, and average corrosion velocity curves, as illustrated in Figure 10, Figure 11 and Figure 12.

As shown in Figure 10, both V-type and Y-type welded joint specimens exhibited higher corrosion rates than the base metal specimens, with the V-type welded joints showing slightly higher corrosion rates than the Y-type joints. The Y-type welded joint specimens underwent different welding procedures and post-weld treatments compared to the V-type specimens, resulting in minor differences in corrosion rates.

Figure 11 reveals the staged characteristics of corrosion kinetics through cross-sectional loss rate data. Unlike the average corrosion rate curves, the base metal specimens showed higher cross-sectional loss rates than the welded joints during the initial corrosion stage (before 9 days). After 9 days, as corrosion progressed, the cross-sectional loss rates of welded joints followed similar trends to their corrosion rates, exceeding those of the base metal. Notably, the performance gap between V-type and Y-type joints observed earlier became less distinct at this stage.

Figure 12 demonstrates that all specimens followed similar corrosion acceleration patterns: the corrosion rates increased progressively with exposure time, while the rate of corrosion acceleration gradually decreases with prolonged exposure time. Welded joint specimens consistently exhibited higher corrosion rates than the base metal, with V-type joints maintaining higher rates than Y-type joints throughout the test.

Synthesizing the results from Figure 10 and Figure 12, the corrosion process of Q500 qENH weathering steel in simulated plateau atmospheric conditions can be divided into two distinct phases: rapid corrosion (0–18 days) and slow corrosion (after 18 days). Throughout identical exposure periods, the base metal consistently demonstrated lower corrosion rates than welded joints. The performance gap between them initially widened during early corrosion stages before stabilizing at later phases.

### 3.3. Corrosion Kinetics

The regression analysis of simulated test results for complex atmospheric pollution environments in plateau regions reveals that the corrosion kinetic model of steel follows a power function variation pattern [30,31]. The corrosion kinetic model is expressed as Equation (1):

(1) $$D=At^{n}$$

where D represents the corrosion depth (unit: mm); t denotes the corrosion time (unit: d); and n indicates the corrosion trend of the specimen.

**Figure 13:**
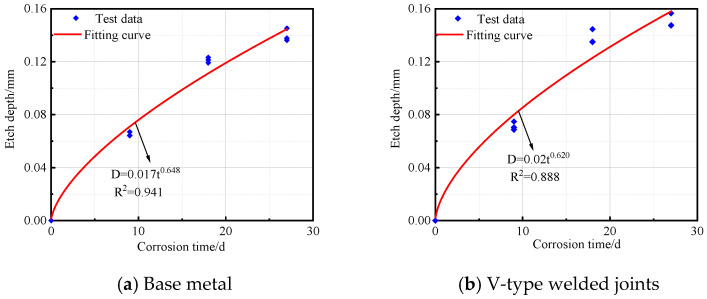

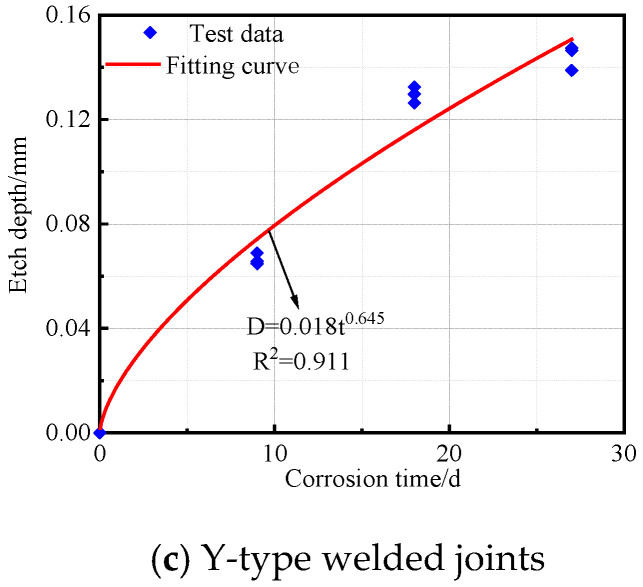
Corrosion kinetic curve of Q500 qENH specimen.

The corrosion depth and time data for the base metal specimens, V-type welded joints, and Y-type welded joints of Q500 qENH weathering steel after corrosion were fitted to obtain the corrosion kinetic curves, as shown in Figure 13. The corresponding fitting equations are given in Equations (2)–(4).

Base metal:

(2) $$D=0.017t^{0.648}\;R^{2}=0.941$$

V-type welded joints:

(3) $$D=0.02t^{0.620}\;R^{2}=0.888$$

Y-type welded joints:

(4) $$D=0.018t^{0.645}\;R^{2}=0.911$$

As shown in R^2^, all three types of specimens demonstrated an excellent fitting performance in their corrosion kinetic curves during the cyclic immersion corrosion test. The corrosion depth exhibited a power function relationship with corrosion time. Furthermore, the variation pattern of corrosion depth over time was highly consistent with the trends observed in both corrosion rate and corrosion speed.

### 3.4. Corrosion Equivalent Conversion

The core steps involved in corrosion equivalent time conversion are [32] based on the equivalent corrosion depth, where $D=D^{\prime }$. The power function model (Equation (1)) is logarithmically transformed. When the periodic immersion corrosion test time equals the equivalent service years of natural atmospheric exposure corrosion, the original equation is rewritten as an expression for natural atmospheric exposure corrosion time T (years) in terms of the periodic immersion corrosion time t (days), as shown in Equation (5):

(5) $$\mathrm{lg}T=(\mathrm{lg}(A/A^{\prime })+n\mathrm{lg}t)/n^{\prime }$$

The results of the 27-day periodic immersion corrosion test are approximately equivalent to 30 years of natural atmospheric corrosion in Beijing. In plateau regions, such as Lhasa, the corrosion rate of steel is approximately 1/8 of that in the Beijing area [33]. The equivalent conversion results of the periodic immersion tests reveal that the equivalent corrosion service years of steel in polluted environments of plateau regions far exceed the 30-year threshold.

## 4. Mechanical Properties Test Results

### 4.1. Fracture Morphology and Failure Mechanism

#### 4.1.1. Tensile Fracture Analysis of Base Metal Specimen

**Figure 14:**
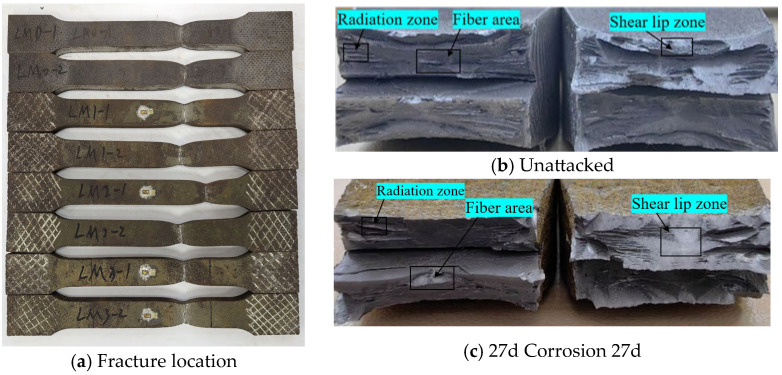
Tensile fracture morphology of base material specimen.

Statistical analysis was conducted on the fracture location and morphology of tensile-fractured specimens from Q500 qENH weathering steel base metal, with the fracture characteristics illustrated in Figure 14.

The tensile fracture of Q500 qENH weathering steel base metal specimens consistently occurred within the parallel section at the middle of the specimens, indicating that corrosion had minimal influence on the fracture location of the base metal specimens. After 27 days of corrosion, the fracture surface exhibited uniform contraction with distinct necking phenomena. The fracture cross-section displayed a stepped morphology accompanied by burrs, demonstrating typical ductile fracture characteristics (Figure 14). This indicates that the failure mode of the weathering steel base metal specimen was minimally affected by corrosion.

#### 4.1.2. Tensile Fracture Analysis of Welded Joint Specimen

**Figure 15:**
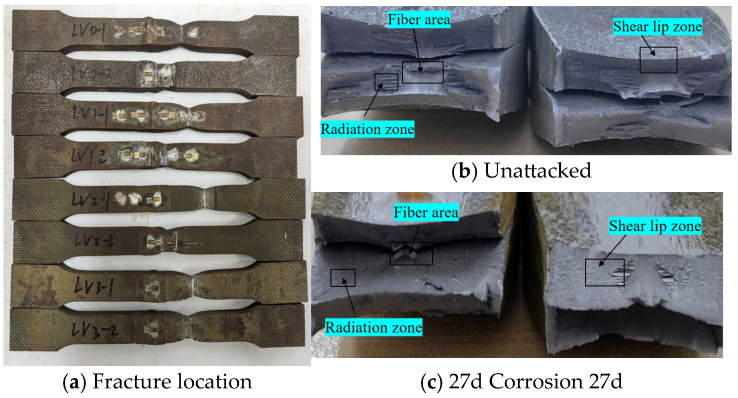
Tensile fracture morphology of V-type welded joint specimen.

**Figure 16:**
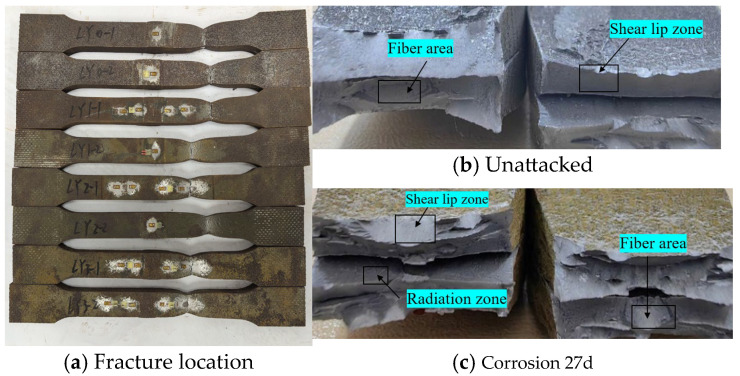
Tensile fracture morphology of Y-type welded joint specimen.

Statistical analysis was performed on the fracture locations and morphologies of the tensile fracture specimens from V-type and Y-type welded joints. The fracture locations and morphologies are shown in Figure 15 and Figure 16.

For both V-type and Y-type welded joint specimens, fractures predominantly occurred in the base metal region near the weld zone. The fracture surfaces exhibited significant necking, along with the presence of fibrous zones and shear lips. The fracture cross-sections displayed a stepped fracture pattern, indicating a typical ductile fracture morphology (Figure 15 and Figure 16). This suggests that corrosion had minimal influence on the fracture locations and failure modes of the V-type and Y-type welded joint specimens, demonstrating that these joints can achieve the original working performance of the weathering steel base metal.

In some V-type welded joint specimens, fractures occurred diagonally from the weld toe into the base metal region. The necking phenomenon gradually diminished with increasing corrosion exposure time, and the fracture mode progressively shifted toward brittle fracture. Since the tensile fractures occurred in the base metal region, in accordance with the specification [34], the weld connection strength is deemed to meet the required standards.

### 4.2. Nominal Stress-Strain Analysis

#### 4.2.1. Nominal Stress–Strain Analysis of Base Metal Specimen

**Figure 17:**
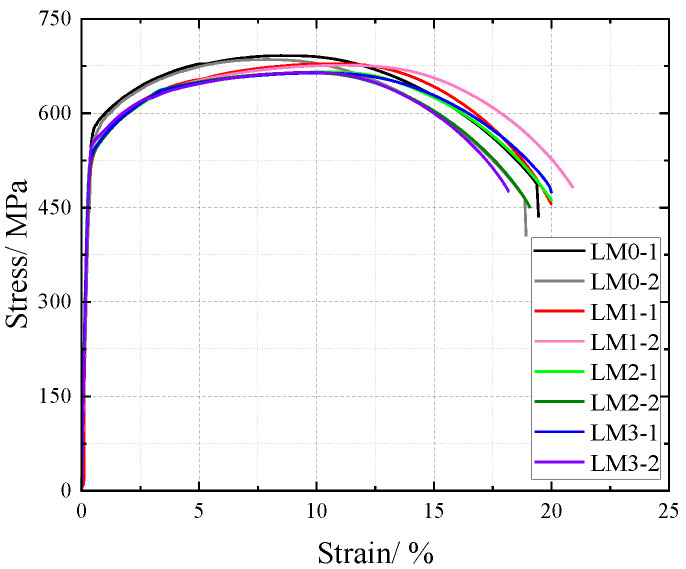
Nominal stress–strain curve of base material specimen after corrosion.

Monotonic tensile tests were conducted at ambient temperature on rust-damaged Q500 qENH weathering steel base metal specimens, with corresponding mechanical response data recorded. The analysis incorporated both the sectional weakening effect induced by corrosion solution on the base metal and the effect of SO_2_ on corrosion. The original cross-sectional area (prior to corrosion) was adopted for stress computation to derive nominal stress data. This approach enabled the plotting of nominal stress–strain curves for base metal specimens under varying corrosion levels, as illustrated in Figure 17.

For the Q500 qENH weathering steel base metal specimens, the nominal stress–strain curves demonstrate that stress decreases progressively with increasing corrosion cycles under identical strain conditions. The stress–strain relationship exhibits distinct elastic and strengthening phases, without a pronounced yield plateau. Within the 10% strain range, a clear demarcation is observed between the nominal stress–strain curves of uncorroded specimens and those subjected to corrosion. However, for the corroded base metal specimens, the nominal stress tends to converge with prolonged corrosion exposure, showing no significant reduction. This behavior indicates that corrosion reduces the effective cross-sectional area of the weathering steel specimens, forming a corrosion-induced rust layer on the surface, which diminishes the tensile strength. Nevertheless, after prolonged exposure, the densified rust layer effectively shields the underlying steel from further corrosive attack.

#### 4.2.2. Nominal Stress-Strain Analysis of Welded Joint Specimen

**Figure 18:**
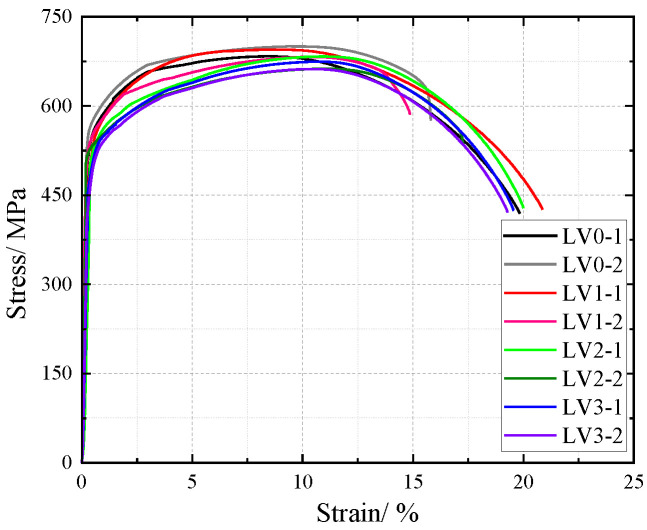
Nominal stress–strain curve of V-type welded joint specimen after corrosion.

**Figure 19:**
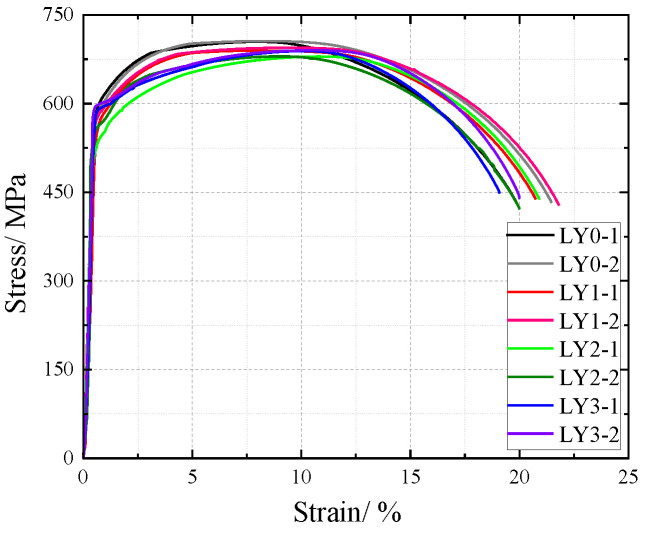
Nominal stress–strain curve of Y-type welded joint specimen after corrosion.

The influence of NaHSO3 corrosion solution on the cross-sectional weakening of V-type and Y-type welded joint specimens made of weathering steel, including the accelerated corrosion rate caused by weld heterogeneity and other corrosion effects, was incorporated as a factor affecting the tensile mechanical properties. The original cross-sectional area of specimens before corrosion was used for stress calculation to obtain nominal stress–strain curves. The nominal stress–strain curves of V-type and Y-type welded joint specimens are shown in Figure 18 and Figure 19, respectively.

The nominal stress of V-type and Y-type welded joint specimens gradually decreases with increasing corrosion cycles under identical strain conditions. Their stress–strain curves exhibit distinct elastic and strengthening stages without a pronounced yield plateau.

The comparative analysis of the nominal stress–strain curves in Figure 17, Figure 18 and Figure 19 reveals that the strain corresponding to the maximum stress in base metal specimens consistently exceeds that of welded specimens. This indicates that material heterogeneity in the weld zone reduces ductility and increases brittleness in weathering steel specimens. The difference in maximum stress values between corroded and uncorroded base metal specimens is smaller than that observed in V-type and Y-type welded joints, demonstrating that welded joints are more susceptible to corrosion effects.

The results demonstrate that corrosion degrades the tensile mechanical properties of weathering steel base metal specimens. Moreover, V-type and Y-type welded joint specimens exhibit greater corrosion sensitivity due to material heterogeneity, leading to more severe cross-sectional weakening. Consequently, the coupling effect of corrosion and welding causes more significant deterioration in the tensile mechanical properties of weathering steel specimens.

## 5. Effect of Corrosion on Tensile Mechanical Properties of Specimens

### Analysis of Yield Strength and Tensile Strength of Specimens

**Figure 20:**
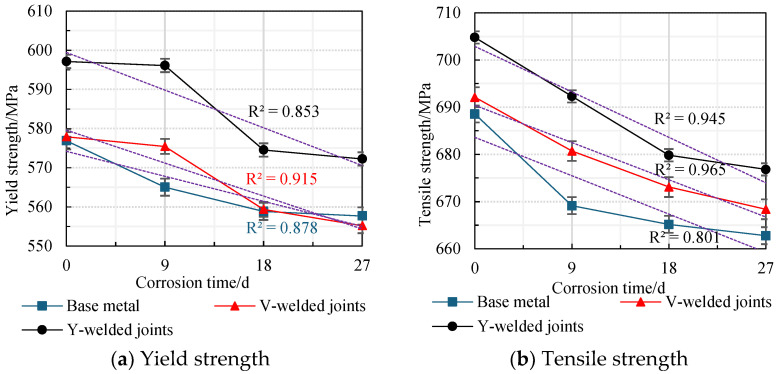
Effect of corrosion time on the strength degradation of specimens.

**Figure 21:**
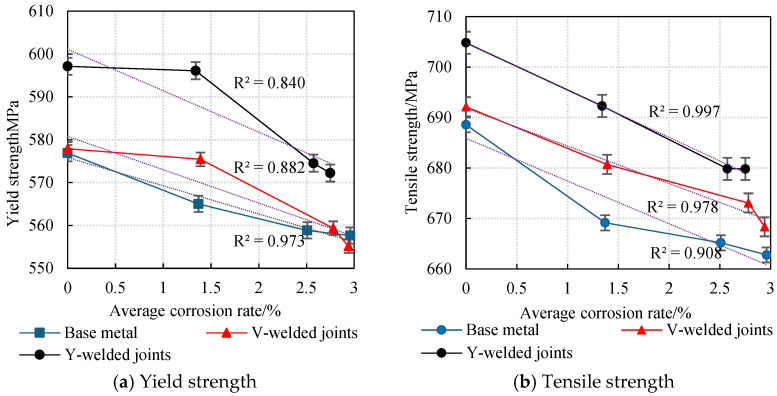
The effect of average corrosion rate on the degradation of specimen strength.

The average values obtained from two parallel specimens per corrosion cycle were plotted as line graphs, revealing the variation patterns in the yield strength and tensile strength of weathering steel standard specimens with corrosion cycles and average corrosion rates, as shown in Figure 20 and Figure 21.

An analysis of Figure 20 and Figure 21 reveals that during the initial corrosion stage (0–9 days), the rapid penetration of the corrosive medium (NaHSO_3_) accelerates corrosion. In the weathering steel base metal specimens, extensive pitting corrosion forms on the surface, while initial defects develop internally, inducing stress concentration. Consequently, both the yield strength and tensile strength degrade rapidly with increasing corrosion duration, with degradation rates of 1.32 MPa/d and 2.16 MPa/d, respectively.

For the V-type welded joint specimens, the degradation rates of the yield strength and tensile strength are 0.27 MPa/d and 1.27 MPa/d, respectively. In contrast, the Y-type welded joint specimens exhibit degradation rates of 0.11 MPa/d and 1.39 MPa/d, respectively.

With a prolonged corrosion duration (9–18 days), the rust layer on the weathering steel base metal specimens gradually densifies, resulting in a reduction in the degradation rates for yield strength and tensile strength to 0.69 MPa/d and 0.44 MPa/d, respectively.

For the welded joint specimens, the heterogeneous material distribution in the weld zone leads to the accelerated degradation of mechanical properties. Specifically, the V-type welded joint specimens exhibit increased degradation rates of 1.79 MPa/d (yield strength) and 0.85 MPa/d (tensile strength), while the Y-type welded joint specimens show further elevated degradation rates of 2.40 MPa/d (yield strength) and 1.38 MPa/d (tensile strength).

When the corrosion duration reached 18–27 days, the rust layers on all weathering steel specimens progressively densified. For the base metal specimens, the degradation rates of yield strength and tensile strength were measured at 0.13 MPa/d and 0.27 MPa/d, respectively, representing reductions of 89.59% and 87.28% compared to the initial corrosion stage (9 days).

The V-type welded joint specimens exhibited degradation rates of 0.47 MPa/d (yield strength) and 0.52 MPa/d (tensile strength), corresponding to decreases of 73.74% and 38.82% relative to the intermediate corrosion stage (18 days). Similarly, the Y-type welded joint specimens demonstrated degradation rates of 0.26 MPa/d (yield strength) and 0.33 MPa/d (tensile strength), showing substantial reductions of 89.17% and 76.09% compared to the 18-day measurements.

The filler wire used for the welded joints was a high-strength low-alloy (HSLA) steel wire. Its designed composition (e.g., the content of alloying elements such as Cr, Ni, Cu) was slightly higher than that of the base metal, aiming to enhance the mechanical properties of the weld zone in the specimen through alloying. During the welding process, the weld zone formed by the fusion of the wire material and the base metal. After uniform fusion, the strengthening effect in the weld zone surpassed that of the base metal, resulting in the weld zone itself exhibiting higher tensile strength than the base metal.

A comprehensive analysis reveals that the rust layer on the Q500 qENH weathering steel specimens progressively densifies with increasing corrosion cycles. Concurrently, the tensile strength and yield strength of the specimens gradually degrade as the corrosion cycles increase. However, the progressive densification of the rust layer results in a gradual decrease in the degradation rate. Specimens with welded joints (particularly those with Y-type welded joints) exhibit higher strength than the base metal.

## 6. Conclusions

This study conducted cyclic immersion accelerated corrosion tests on Q500 qENH weathering steel base metal specimens, V-type welded joints, and Y-type welded joints to simulate the corrosion process under complex atmospheric pollution conditions in plateau regions. Monotonic tensile tests were subsequently performed, yielding the following conclusions:

(1) With prolonged corrosion exposure, the number of surface pitting pits on the specimens progressively increased. Macroscopic corrosion morphology analysis revealed that the rust layer evolved from yellowish-brown with black corrosion products to granular iron oxides, followed by progressive thickening and eventual spalling. By 18 days of corrosion, the rust layer had become exposed across most specimen surfaces, with the inner rust layer exhibiting significantly enhanced densification. Consequently, the corrosion rate stabilized.
(2) The corrosion process can be divided into two distinct stages: a rapid corrosion stage (before 18 days), and a slow corrosion stage (after 18 days). For both types of welded joint specimens, the corrosion rates were consistently higher than those of the base metal specimens under identical corrosion durations. Moreover, the difference in corrosion rates between the base metal and welded joint specimens initially increased, subsequently decreased, and eventually stabilized over time.
(3) Regression analysis revealed that the corrosion kinetics of the steel specimens followed a power–law relationship (*D* = *At^n^*), with the corrosion depth of all three specimen types exhibiting good fitting accuracy to this model. The derived power–law relationship was consistent with the observed trends in both corrosion rate and degradation rate. The equivalent conversion between periodic immersion corrosion tests and natural atmospheric corrosion in polluted environments was established. The results demonstrate that the 27-day accelerated corrosion test significantly exceeded the 30-year corrosion threshold under natural atmospheric pollution conditions in plateau regions. This conclusively validates the superior service life of Q500 qENH weathering steel in such environments.
(4) After 27 days of corrosion, partial fracture surfaces of V-type welded joint specimens appeared at the weld toe, exhibiting brittle fracture characteristics. The remaining specimens demonstrated ductile fracture behavior. The material heterogeneity in both V-type and Y-type welds showed no significant influence on the microstructural morphology of weathering steel specimens, nor did it alter their fracture modes.
(5) With prolonged corrosion exposure, all weathering steel specimens exhibited a progressive degradation in yield strength, yield load, tensile strength, and ultimate load, with gradually decreasing degradation rates. After 27 days of corrosion, the base metal specimens, V-type, and Y-type welded joint specimens showed yield strength reductions of 3.33%, 3.93%, and 4.17%, respectively. The material inhomogeneity in weld zones was found to adversely affect the corrosion resistance of weathering steel specimens. However, due to the presence of the weld metal alloy, the welded joint specimens exhibit higher strength than the base metal specimens.

## Figures and Tables

**Table 1:** Chemical element content of Q500 qENH steel.

Chemical Composition/%	C	V	P	Mn	S	Cr	Mo	Ni	Cu	Nb	Si
Q500 qENH	0.060	0.036	0.013	1.360	0.003	0.470	0.100	0.410	0.250	0.025	0.340

**Table 2:** Chemical element content of welding electrode for Q500 qENH steel welded joint.

Chemical Composition/%	Si	C	Mn	S	P	Cr	Ni	Mo	Cu
JWER60NHQ	0.360	0.050	1.130	0.002	0.008	0.360	0.550	—	0.340
JWS60NHQ	0.380	0.043	1.610	0.003	0.013	0.330	0.420	—	0.280

**Table 3:** Grouping of corrosion specimens.

Corrosion Cycle /d		Corrosion Specimen	
\d	Base Metal	V-Type Welded Joints	Y-Type Welded Joints
0	LM0-1, LM0-2, LM0-3	LV0-1, LV0-2, LV0-3	LY0-1, LY0-2, LY0-3
9	LM1-1, LM1-2, LM1-3	LV1-1, LV1-2, LV1-3	LY1-1, LY1-2, LY1-3
18	LM2-1, LM2-2, LM2-3	LV2-1, LV2-2, LV2-3	LY2-1, LY2-2, LY2-3
27	LM3-1, LM3-2, LM3-3	LV3-1, LV3-2, LV3-3	LY3-1, LY3-2, LY3-3
Total/block	12	12	12

## Data Availability

The original contributions presented in this study are included in the article. Further inquiries can be directed to the corresponding author.
